# Epidemiology and Clinicopathological Profile of Renal Cell Carcinoma: A Review from Tertiary Care Referral Centre

**DOI:** 10.15586/jkcvhl.2021.154

**Published:** 2021-01-20

**Authors:** Likhiteswer Pallagani, Gautam Ram Choudhary, Pandey Himanshu, Vijay K.S. Madduri, Mahendra Singh, Prateek Gupta, Nikita Shrivastava, Gaurav Baid, Rao Meenakshi, Nalwa Aasma, Puneet Pareek, Misra Sanjeev

**Affiliations:** 1 Department of Urology, All India Institute of Medical Sciences, Jodhpur, India;; 2 Department of Pathology, All India Institute of Medical Sciences, Jodhpur, India;; 3 Department of Surgical Oncology, All India Institute of Medical Sciences, Jodhpur, India;; 4 Department of Radio-Therapy, All India Institute of Medical Sciences, Jodhpur, India

**Keywords:** epidemiology, laparoscopy, minimally invasive surgery, paraneoplastic syndromes, renal cell carcinoma, robotic surgery, Western India

## Abstract

Renal cell carcinoma (RCC) accounts for 3% of all adult cancers and 85% of all kidney tumours. Incidence of RCC is lower in Asian region, particularly in India, probably due to lack of reporting. Most of the data about RCC are from Western countries; and data from India are scarce, especially regarding para-neoplastic syndromes. We sought to determine the epidemiology, clinicopathological profile and management of RCC in a tertiary care centre in Western India.

This was a retrospective study that involved data analysis of records of RCC patients who presented to our institution from April 2016 to February 2020. Laboratory investigations, including tests for paraneoplastic syndrome (PNS), and relevant radiologic investigations were performed and treatment was offered according to the stage, patient factors and available modalities.

A total 142 RCC patients were included in the study. The median age of presentation was 58 years. Most of the patients (67%) were symptomatic, and 33% of the patients were asymptomatic, and the RCC was diagnosed incidentally. A large number of patients (56.3%) had PNS. The most common histopathologic type of RCC was clear cell carcinoma (68.8%), followed by papillary (20%) and chromophobe (8%) carcinoma. 40% of carcinomas with sarcomatoid differentiation were seen in patients under 50 years of age. Two cases of multicystic RCC were both seen in patients less than 50 years of age. 65.5% of the patients presented at Stage 1 and 2. Most surgeries (71.2%) were done in a minimally invasive manner.

A significant number of patients were asymptomatic, in which RCC was detected incidentally. The age of presentation was earlier, yet the patients had a higher tumour stage. More than half of the patients had PNSs. Despite growing trend towards Western data, the significantly higher number of patients with PNSs and early age of presentation suggest inherent differences in tumour biology, possibly related to differences in genetic and environmental factors.

## Introduction

Renal cell carcinoma (RCC) accounts for 3% of all adult cancers and 85% of all kidney tumours (1). Incidence of RCC is lower in Asian region, particularly in India, probably owing to lack of reporting (2). The incidence is expected to rise in India due to increasing life expectancy, rising awareness, better diagnostic facilities and growing prevalence of risk factors such as obesity (3). Most of the data about RCC are from Western countries; and data from India are scarce (4). Because the clinical spectrum of a disease may differ across the globe, this study was undertaken with the objectives of studying the demography, presentation and management of RCC at a referral centre located in western part of India, with the ultimate aim to comment on the clinical heterogeneity of the disease, if any, and to fill the knowledge gap among researchers.

## Methods

This is a retrospective study of prospectively kept record, which involved data analysis of records of RCC patients who presented to our institution from April 2016 to February 2020. All the patients had either contrast-enhanced computerised tomography (CECT) scans or magnetic resonance imaging, where renal CECT was contraindicated for clinical staging and characterisation of the renal tumour. Laboratory investigations, including tests for paraneoplastic syndrome (PNS), were performed, and treatment was offered according to the stage, patient factors and available modalities. Renal masses diagnosed as benign were excluded from the analysis. The seventh and eighth edition of the American Joint Committee on Cancer tumour, nodes and metastasis (TNM) staging systems were used to classify cancer stage and grade (5); the seventh edition for cases managed before January 2018 and the eighth edition for cases managed after January 2018. The histological subtypes were classified as per the classification of World Health Organization for renal tumours, 2016 (6).

## Results

**Figure 1:**
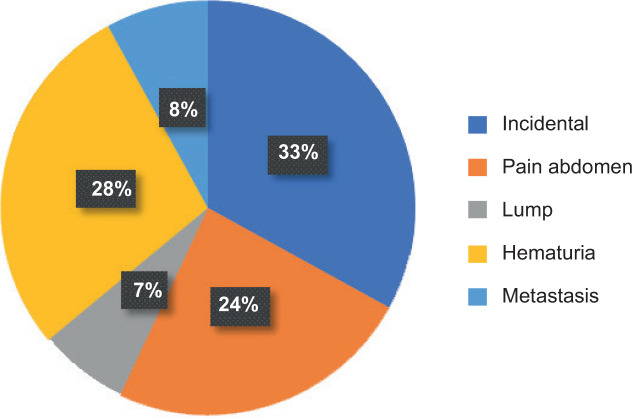
Presentation.

**Figure 2:**
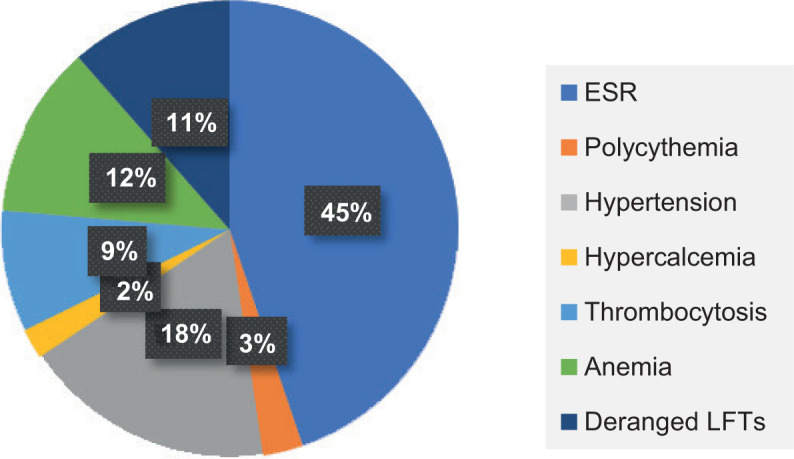
Paraneoplastic syndrome.

**Figure 3:**
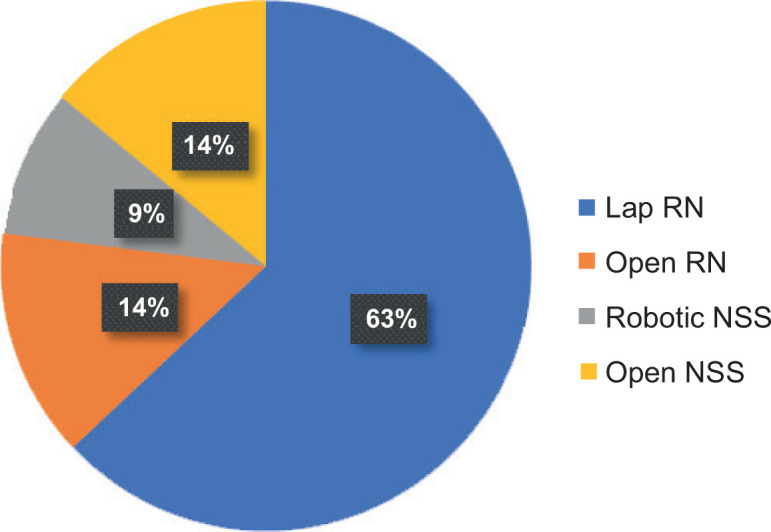
Surgeries.

A total of 142 patients vising the institute were diagnosed with RCC, of which 104 patients were male and 38 patients were female. Most patients presented were in the sixth and seventh decades, 43 patients (30.2%) were below 50 years of age. 58 years being the median age at presentation, the youngest patient was 20-year-old and the oldest was 84-year-old (Table 1). Right- and left-sided RCC were equally distributed, and three patients had bilateral tumours. In our study, in many patients, the renal mass was detected incidentally (34%). In symptomatic patients, haematuria (29%) and abdominal pain (24%) were the most common complaints (Figure 1). Nearly 25.6% of the patients had comorbidities, hypertension (HTN) (n = 22) and diabetes mellitus (DM) (n = 17) being the most common (Table 2). PNSs were seen in 56.3% (n = 80) of the patients, with raised erythrocyte sedimentation rate (ESR) (n = 50) being the most common PNS (Figure 2). At presentation, 106 (74.6%) patients were treatment naïve, 31 (21.8%) patients had undergone radical or partial nephrectomy, and 5 (3%) patients had received tyrosine kinase inhibitor (TKI) elsewhere before coming to our centre. Of the 106 treatment naïve patients, 97 underwent surgery as their initial mode of treatment, 9 received TKI upfront and 2 received TKI after cytoreductive nephrectomy. None of the patients who received TKI upfront were deemed fit for any surgical intervention later on. Radical nephrectomy was done in 73 patients, nephron sparing surgery (NSS) in 21 patients and cytoreductive nephrectomy in 3 patients. Most of the radical nephrectomies in our centre were minimally invasive, with conversion to open procedure in three cases. Only cases that were more than 20 cm or involving the adjacent organs or with inferior vena cava (IVC) involvement (level 2) were planned for open surgeries. NSS done in the initial years were open surgeries, but as our institution acquired the da Vinci robotic system, all the NSS in 2019 were robot assisted, of which one was converted to open. The decision to perform NSS was based on the tumour characteristics, patient factors and patient’s choice (Figure 3). Lymph node dissection was done in four cases, of which one case had metastatic deposits. The disease was organ confined (pT1 and pT2) in 93 (65.9%) patients; however, only 26 patients had a tumour size of <4 cm (pT1a). IVC thrombus was seen in 13 patients, most common being level 1 (n = 7), followed by level 2 (n = 4) and level 4 (n = 2). There was no significant difference in clinical stage of the tumour, tumour size, tumour grade, presence of lymph nodes and IVC thrombus in different age groups (Table 3). Of the 106 patients who were treatment naïve, the most common histopathologic type of RCC was clear cell carcinoma, comprising 73 (68.8%) cases, followed by papillary (20%) and chromophobe carcinoma (8%). The other rare tumours were peripheral neuroectodermal tumour (PNET) (n = 2), tubulocystic RCC (n = 1) mucinous tubular and spindle cell carcinoma (n = 1) and solitary fibrous tumour (n = 1) (Table 4). Although clear cell RCC was the most common histologic type in younger age group patients (<50 years), seen in 70% (n = 19) of the patients, the uncommon histologic types were also seen most commonly in this age group. Two patients with sarcomatoid differentiation and two patients with multicystic variant were both <50 years old. Three patients had von Hippel Lindau syndrome, of which two patients had bilateral multiple tumours. One patient with bilateral tumours was managed with cytoreductive nephrectomy and adjuvant TKI and the other patient was managed with upfront TKI and close follow-up. Of total 5 patients with sarcomatoid differentiation, in 2 adjuvant treatment was indicated and chemotherapy (gemcitabine + doxorubicin) was administered, both at 12 months follow-up were recurrence free.

**Table 1:** Age at presentation.

S. No.	Age group	No. of patients (n = 142)
1	0–20	1
2	21–30	3
3	31–40	16
4	41–50	23
5	51–60	42
6	61–70	41
7	71–80	13
8	81–90	2

**Table 2:** Comorbidities.

S. No.	Comorbidities	No. of patients (n = 48)
1	Hypertension	22
2	Diabetes mellitus	17
3	von Hippel Lindau syndrome	2
4	Coronary artery disease	3
5	Chronic obstructive pulmonary disease	2
6	Hypothyroidism	2

**Table 3:** Stage at presentation.

S. No.	Clinical Stage	No. of patients (n = 142)
1	T1a	26
2	T1b	31
3	T2a	27
4	T2b	10
5	T3	19
6	T4	12
7	Metastasis	17

**Table 4:** Histopathology in patients <50 years of age.

S. No.	Type of RCC	Number (n = 25)
1	Clear cell carcinoma	13
2	Sarcomatoid variant of clear cell carcinoma	2
3	Multicystic variant of clear cell carcinoma	2
4	Papillary carcinoma	3
5	Chromophobe carcinoma	2
6	Tubulocystic RCC	1
7	PNET of kidney	1
8	Solitary fibrous tumour	1

## Discussion

According to available literature, RCC is a disease of elderly population (7). Although data from a developing country like India are limited, as per the SEER database, almost 50% patients with RCC present in the age group between 55 and 75 years and the median age at presentation is 64 years (8). The median age of presentation in this study was 58 years, with one-fourth (26.7%) of the patients less than 50 years of age, and 14% below 40 years of age. This shows a much younger age of presentation in this area. These findings are similar to other Indian studies and Asian studies (4, 9–14). Asian population has a reportedly low incidence of RCC, which may be multifactorial, including genetic and environmental factors or other factors like low reporting (15). The younger age of presentation may also be attributable to environmental factors, dietary factors or genetic susceptibility, which needs to be conclusively addressed by larger epidemiological studies (16). As per existing literature from developed world, the male to female ratio of RCC patients is 2:1 (17). We found an even higher incidence of renal cancers in males, with a male to female ratio of 2.7:1. This finding is similar to that of other Indian studies (2.9) and Asian countries (13, 18). This may be due to the lower incidence of smoking among women or low socioeconomic conditions, leading to difference in treatment seeking behaviour (19).

In our study, most of the patients were symptomatic (67%) with most common presenting symptom being haematuria (28%) followed by pain abdomen (24%), and incidentally diagnosed RCC accounting for only 33%. This is in contrast to findings in Western studies where more than 60% of renal cancers are diagnosed incidentally (20). The incidence of incidental RCC is still higher in our data compared with other Indian studies (4). Similar findings have been noted in another Indian study (21). This could be due to the changing trend towards earlier detection of RCC in India.

It has been established in literature that approximately 20% of patients with RCC develop PNSs (22, 23). These data have predominantly been derived from Western population. In our study, 56.3% patients developed PNS, with raised ESR being the most common (62.5%), followed by hypertension (25%), anaemia (17%), deranged LFTs (16%), thrombocytosis (12%), polycythaemia (4%) and hypercalcaemia (3%). The postulated mechanisms for PNS in RCCs are elevated cytokines (especially IL-6), tumour secreting hepatotoxins and lysozymes (hepatic dysfunction/Stauffer syndrome), renin and IL-6 secretion by tumour cells (hypertension), erythropoietin secretion by tumour cells (polycythaemia), thrombopoietin secretion by tumour cells (thrombocytosis), chronic disease, poor nutritional status and increased iron-binding protein Lactoferrin (anaemia) (21). Immunohistochemistry (IHC) is not routinely applied to the tumour slides for demonstration of tumour cell production of the paraneoplastic agent. Interestingly, hypercalcaemia, which has been noted to be a common PNS in RCC, was found to be the least common in our study (15, 24). This study is the first that highlights the incidence of PNS in the Indian population, and the figure is significantly higher. The reason for the high incidence of PNS could be higher stage of presentation in Indian population, or it could be due to genetic or environmental factors, which need to be further studied.

With respect to histopathology and stage, there are varied data published in the Indian population. The data of histopathology from Western countries show clear cell RCC to be the most common variant, accounting for close to 85% (25, 26). In our study, clear cell RCC accounted for only 68.8% of all tumours. In patients less than 50 years of age, the multicystic variant was more common than in older patients. Moreover, 40% of the patients with sarcomatoid differentiation (2/5) were also <50 years of age. These findings are in variance from those of other Indian studies (4, 10), and from most of the Asian studies (12, 18), which show similar findings as those from the Western population. Only one other Indian study shows similar findings (9). In addition, in our study population, younger patients had varied histological pattern, with higher chances of aggressive carcinoma. Stage at presentation in our patients was also different from those in the West. SEER data suggest that 60–70% of their patients present at Stage 1 (8), but in our study only 40% cases presented at Stage 1 and 65.5% of patients presented at Stages 1 and 2, this shows an advanced stage at presentation of patients with RCC in India compared with Western counterparts where they present at a much earlier stage. This is also reflected in the lower proportion of partial nephrectomies done in this study. Interestingly though, the proportion of patients presenting with localised carcinoma is much higher in this study that other Indian studies (4, 10). This could be because of increased imaging performed in recent years, with widespread use of cross-sectional imaging.

The number of radical nephrectomies done in this study far outweighs the NSS. This is probably due to the higher stage of presentation in our population. Laparoscopic radical nephrectomies were the standard of care rather than exception in our study. Our experience with the laparoscopic surgery has been positive with low open conversion rate (5%) and low morbidity from surgeries. Although the traditional indications for laparoscopic radical nephrectomy are for localised carcinoma (size up to 10–12 cm) (14), we believe there is further scope for expanding these indications to t2b and t3a tumours. Open surgeries still play a role in difficult nephrectomies, for T3b and T3c tumours, and large RCC >15 cm in maximum dimension where placing ports can be difficult; but the indications for it are shrinking day by day. Management of nonlocalised RCC and syndromic patients was discussed in multidisciplinary team and TKI and chemotherapy were used as neo-adjuvant, adjuvant or only treatment modality, according to disease and patient factors.

The limitations of this study are that it was a retrospective study, with smaller number of patients, a few of the patients were operated elsewhere and came to our institution for further management and/or follow-up, and detailed follow-up was not available.

## Conclusion

In our study, a significant number of patients were asymptomatic, in which RCC was detected incidentally. The age of presentation was earlier, yet the patients had a higher tumour stage. More than half of the patients had PNS. Clear cell RCC was the most common histologic type, though less common than that is reported in literature. More number of patients less than 50 years of age had sarcomatoid differentiation, with unusual presentations being more common. In conclusion, the findings suggest a growing trend towards Western data. However, the significantly higher number of patients with PNS suggests inherent differences in tumour biology, possibly related to differences in genetic and environmental factors.
